# Impact of Remote Patient Monitoring Systems on Nursing Time, Healthcare Providers, and Patient Satisfaction in General Wards

**DOI:** 10.7759/cureus.61646

**Published:** 2024-06-04

**Authors:** Pavithra L S, Sheen Khurdi, Priyanka T G, Patrisia Mary S

**Affiliations:** 1 Hospital Administration, M.S. Ramaiah Memorial Hospital, Bengaluru, IND; 2 Medical Surgical Nursing, M.S. Ramaiah Memorial Hospital, Bengaluru, IND

**Keywords:** general wards, healthcare efficiency, nursing workload, patient safety, remote patient monitoring systems

## Abstract

Introduction: Remote patient monitoring systems (RPMS) are increasingly integrated into hospital wards to improve patient safety and reduce the workload on healthcare professionals (HCPs). This study evaluates the efficacy of RPMS in general wards, focusing on their impact on nursing efficiency, patient care, HCPs, and patient satisfaction.

Methods: A comprehensive time-motion study was conducted along with surveys targeting HCPs and patients in M.S. Ramaiah Memorial Hospital, Bangalore, India, which has implemented RPMS in general wards. The study involved observing and comparing nursing activities in RPMS-equipped wards versus control wards without RPMS across various shifts. In addition, feedback on the system's impact on patient safety, overall care quality, and usability was gathered through a survey form.

Results: RPMS decreases the amount of time nurses spend on routine monitoring, communication, and coordination, enabling a 43.11% increase in time available for patient care. More than 89% of HCPs noted improvements in the level of care and overall patient safety. More than 80% of the HCPs also noted improvement in the patient’s experience. More than 50% of HCPs find RPMS easy to use and user-friendly. More than 60% of the patients noted an overall improvement in care quality.

Conclusion: RPMS has proven to be a valuable asset in hospital wards, enhancing patient monitoring and safety while reducing the workload on staff. In addition, significant time savings on routine tasks and high satisfaction levels from both staff and patients underscore the system's benefits.

## Introduction

In general wards, patient monitoring typically occurs intermittently [1], unlike the continuous monitoring found in intensive care units (ICUs) [2]. Due to this gap, signs of health deterioration are often missed [3], which leads to delayed intervention compromising patient safety [4]. Continuous monitoring technologies are poised to bridge this disparity, enhancing safety and improving healthcare delivery in wards [5].

Recent advancements in patient monitoring technologies have significantly expanded capabilities beyond the ICUs. Research by Garssen et al. (2023) [6] and Weenk et al. (2020) [7] demonstrates the effectiveness of contact-based wearables for continuous monitoring in general wards. Furthermore, non-contact methods like ballistocardiography, highlighted by Ginsburg et al. (2022) [8] and Wang et al. (2022) [9], offer non-invasive monitoring, increasing patient comfort and compliance. These innovations not only extend the reach of continuous monitoring but also have the potential to improve health outcomes through enhanced preventive care [5,10-15].

Since November 2022, M.S. Ramaiah Memorial Hospital (MSR), Bangalore, India, equipped with over 500 beds, has embraced these technological advancements by implementing a continuous non-invasive remote patient monitoring system (RPMS) in its wards. The real-world impact of this RPMS on patient care, usefulness, ease of use, and satisfaction among healthcare practitioners (HCPs) and patients remains under-explored. This study evaluates the RPMS by conducting a comprehensive time-motion study to assess nursing efficiency and workflow. In addition, surveys targeting HCPs and patients measure the system’s overall impact on patient safety, usefulness, ease of use, and experience. This multifaceted approach aims to deliver detailed insights into the benefits of integrating advanced monitoring technologies in general ward settings, thereby enriching the understanding of their practical implications.

## Materials and methods

RPMS

**Figure 1:**
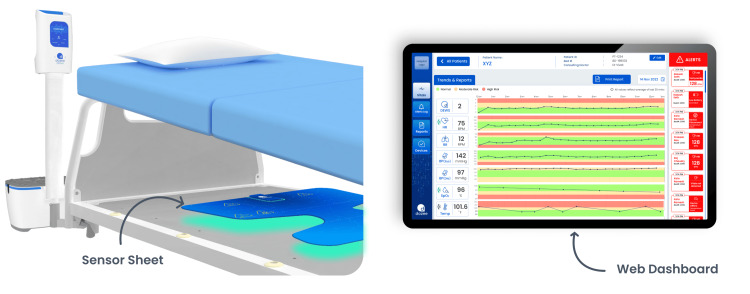
RPMS: The sensor sheet under the bed (left) captures microvibrations from the patient, which are processed to display measured vitals, trends, and alerts on the web dashboard (right). RPMS: Remote Patient Monitoring System

RPMS utilizes artificial intelligence (AI) algorithms combined with ballistocardiography (BCG) data from piezoelectric sensors to enable contactless monitoring of vital signs, including heart rate, respiration rate, blood pressure, and patient movement. Enhanced by both wired and wireless non-invasive accessories for measuring oxygen saturation and temperature, the RPMS at MSR has proven its reliability and accuracy in monitoring vital signs, confirmed by clinical studies [16-19]. In addition, the system features a customizable multi-tiered early warning system (EWS) for assessing severity levels, supported by a user-friendly web dashboard and mobile app for centralized and remote ward monitoring. In addition to the digitization of the vital signs and trends, this RPMS also provides digital reports on these. MSR utilizes this BCG-based RPMS and EWS for efficient patient monitoring, reporting, and informed clinical decision-making (Figure 1). 

Approval

This study received approval from the M.S. Ramaiah Medical College and Hospitals Ethics Committee on November 6, 2023. The approval number is MSRMC/EC/AP-01/11-2023.

Time-motion study

A comprehensive time-motion direct observation study was conducted at MSR to compare nursing activities in wards equipped with RPMS and wards without these systems. The research encompassed 120 hours of observations across morning, evening, and night shifts in a general ward setting with a capacity of 30 beds. The staffing ratios historically in the wards are 1:6 to 1:7 during morning and evening shifts and from 1:6 to 1:9 during night shifts. Each morning and evening shift lasted six hours, while the night shift lasted for 12 hours. The choice of these specific wards and shifts and the total observation hours were guided by the objective to capture a comprehensive range of nursing activities under different working conditions.

Observers followed nurses who had at least 3+ years of experience and were directly involved with patient care, during their duties, which included direct patient care tasks, such as monitoring and charting vitals, administering and recording medications, and addressing general care needs like dietary and bathroom assistance. In addition to these tasks, nurses also assisted during consultant rounds, maintained necessary documentation, and conducted thorough handover sessions at the end of each shift to ensure ongoing, high-quality, and safe patient care. Furthermore, nurses’ responsibilities in managing ward operations, such as ordering supplies, coordinating cleaning, and handling administrative duties, were documented to assess the integration and impact of monitoring technologies on overall ward efficiency.

Data collection was performed using standardized data sheets, which were designed to capture detailed information on time allocation for various activities, ward demographics, the number of patients, the number of on-duty nurses, and nurse demographics. Observers received rigorous training on the procedures of the time-motion study and the proper use of data sheets to ensure consistency and reliability in data recording. Observations were conducted unobtrusively to minimize disruption to ward activities. Confidentiality and privacy of the patient and nurse data were maintained.

For data analysis, the activities were grouped under five major categories: patient care, medication and mobilization, communication and coordination, charting and documentation, spot vitals check, and other activities.

HCP feedback on RPMS

A survey (see Appendix A) was conducted among HCPs, i.e., both nurses and doctors, working in RPMS-equipped wards to get their perspectives on perceived benefits, patient safety and care, ease of use, patient experience, and satisfaction. HCPs were selected to represent a diverse range of experiences, encompassing different shifts and roles within the hospital. Prior to participation, all respondents provided informed consent. To maintain consistency in responses, the majority of questions were identical for both nurses and doctors. The survey format included close-ended questions. It also collected demographic information, with responses anonymized to protect participant confidentiality. Descriptive statistics were used to analyze both the demographic and survey responses.

Patient feedback on RPMS

To evaluate patient perceptions, a survey among patients admitted to the RPMS ward was conducted. Eligible participants were those aged 18-80, literate, conscious, responsive, and had stayed in the RPMS ward for at least one day. The survey instrument, detailed in Appendix B, consisted of closed-ended questions. These questions were designed to quantitatively assess patients' perceptions of care, comfort, mobility, and safety, offering insights into their experiences with the monitoring systems. Informed consent was obtained from all participants involved in the survey. The survey was administered face-to-face by trained nurses.

## Results

Time-motion study

Observations totaling 120 hours were conducted in both control and RPMS wards during morning, evening, and night shifts. Monitoring was distributed evenly with 30 hours each for morning and evening shifts and 60 hours for night shifts. Thirty nurses, split evenly between control and RPMS wards, were observed across these shifts. The nurse-to-patient ratio was consistent across control and RPMS wards (1:6) with slight variations during the evening shift in the RPMS ward (1:7). Nurses in the RPMS ward typically had more experience, especially during evening and night shifts (Table 1).

**Table 1:** Nurse-to-patient ratio and nurse demographics

Shift	Ward	Median nurse-to-patient ratio	Nurse followed	Experience (mean ± SD)	Hours observed
Morning	Control ward	1:6	5	9.4 ± 5.72	30
	RPMS ward	1:6	5	9.8 ± 3.11	30
Evening	Control ward	1:6	5	6.2 ± 3.96	30
	RPMS ward	1:7	5	12.8 ± 2.86	30
Night	Control ward	1:6	5	5.4 ±1.14	60
	RPMS ward	1:6	5	10.3 ± 4.68	60

**Figure 2:**
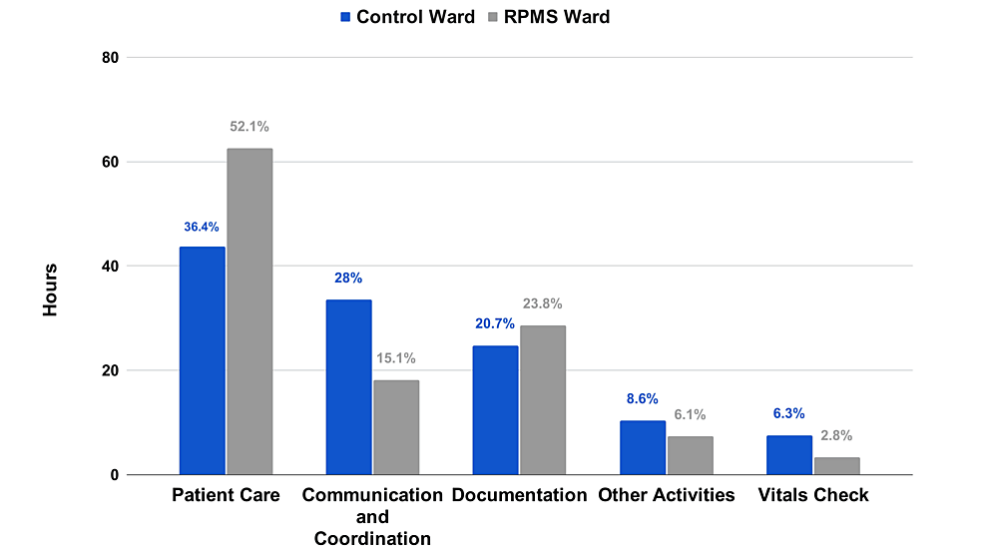
Time spent by the nurses on various activities (combined).

Nurses in the RPMS ward significantly increased their patient care time by 43.11% compared to the control ward. They also required 45.9% less time for communication and coordination. The duration for vital checks was reduced by 55.96%, while documentation activities rose by 15.4%. Time spent on other activities decreased by 29.31% (Figure 2).

**Figure 3:**
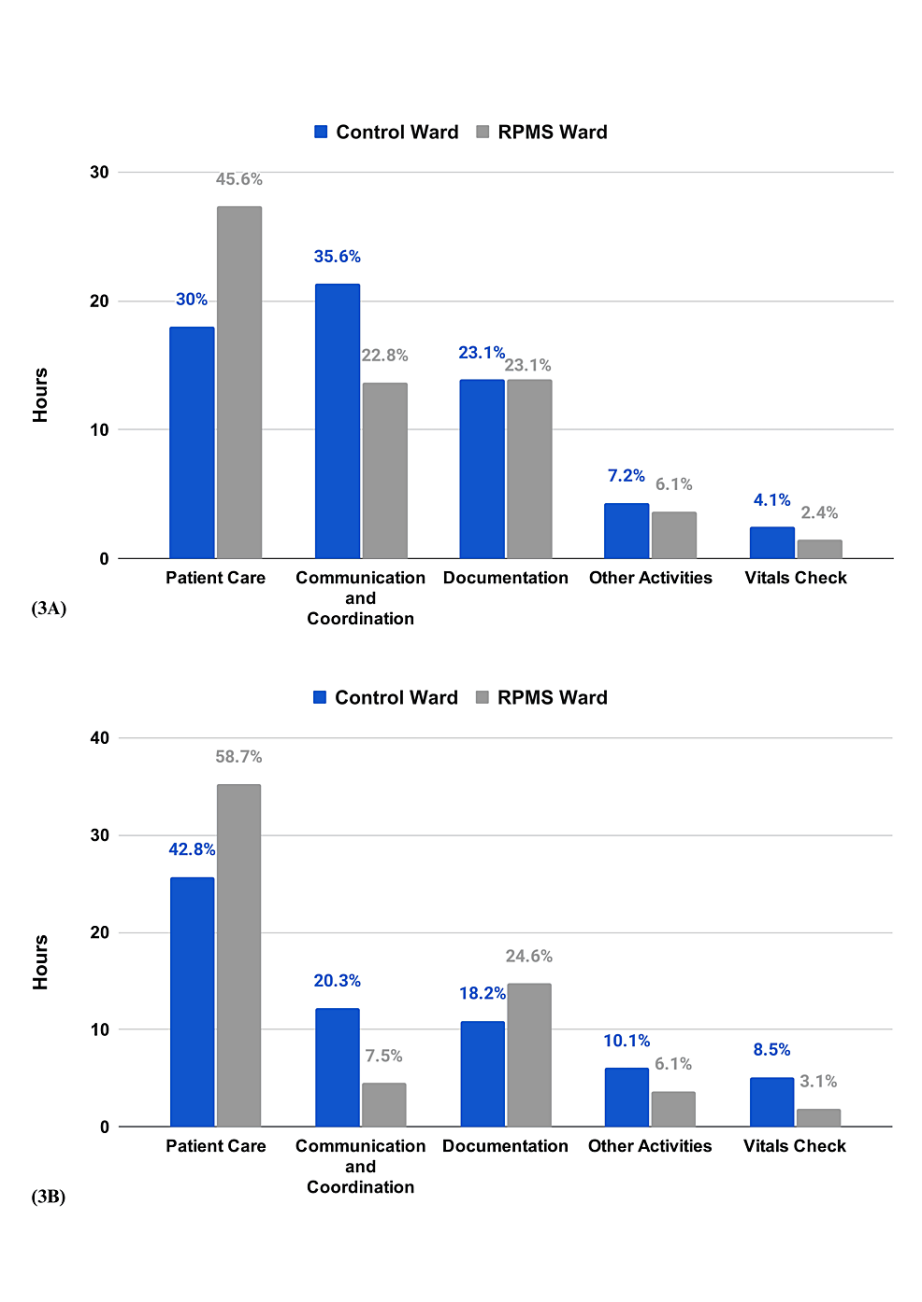
Time spent by the nurses on various activities A) Day B) Night

During the day shift, RPMS ward nurses spent 51.73% more time on patient care than those in the control ward. This trend continued into the night shift with a 37.1% increase. Communication and coordination times decreased by 35.9% during the day and 65.27% at night. Documentation time increased by 35% at night, and vital checks saw reductions of over 40% and 63.4% during day and night shifts, respectively. Time spent on other activities was also reduced by 15.38% during the day and 39.3% at night (Figure 3A, 3B).

HCP feedback

The survey was conducted with a group of 39 nurses and 28 doctors. For the nurses surveyed, the gender ratio heavily favors females. The gender distribution is more balanced among doctors, with a ratio of 18 males to 10 females. The surveyed nurses had more experience compared to the doctors (Table 2).

**Table 2:** HCP Demographics * Data unavailable for 1 Nurse  ** Data unavailable for 1 Doctor

	Nurse	Doctor
Age in years (Range)	31.21* (21-50)	28.33** (23-39)
Experience in years (Range)	7.96* (1-24)	2.2** (0.25-5)
Male:Female	1:38	18:10

RPMS Benefits

**Figure 4:**
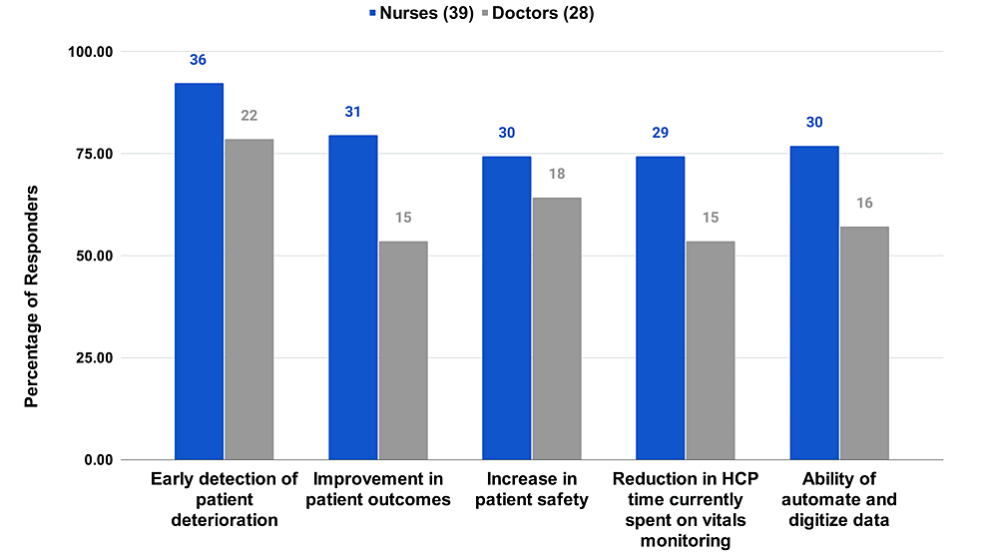
HCP survey questions on the RPMS Benefits In your opinion what are the benefits of using continuous vitals monitoring systems?

**Figure 5:**
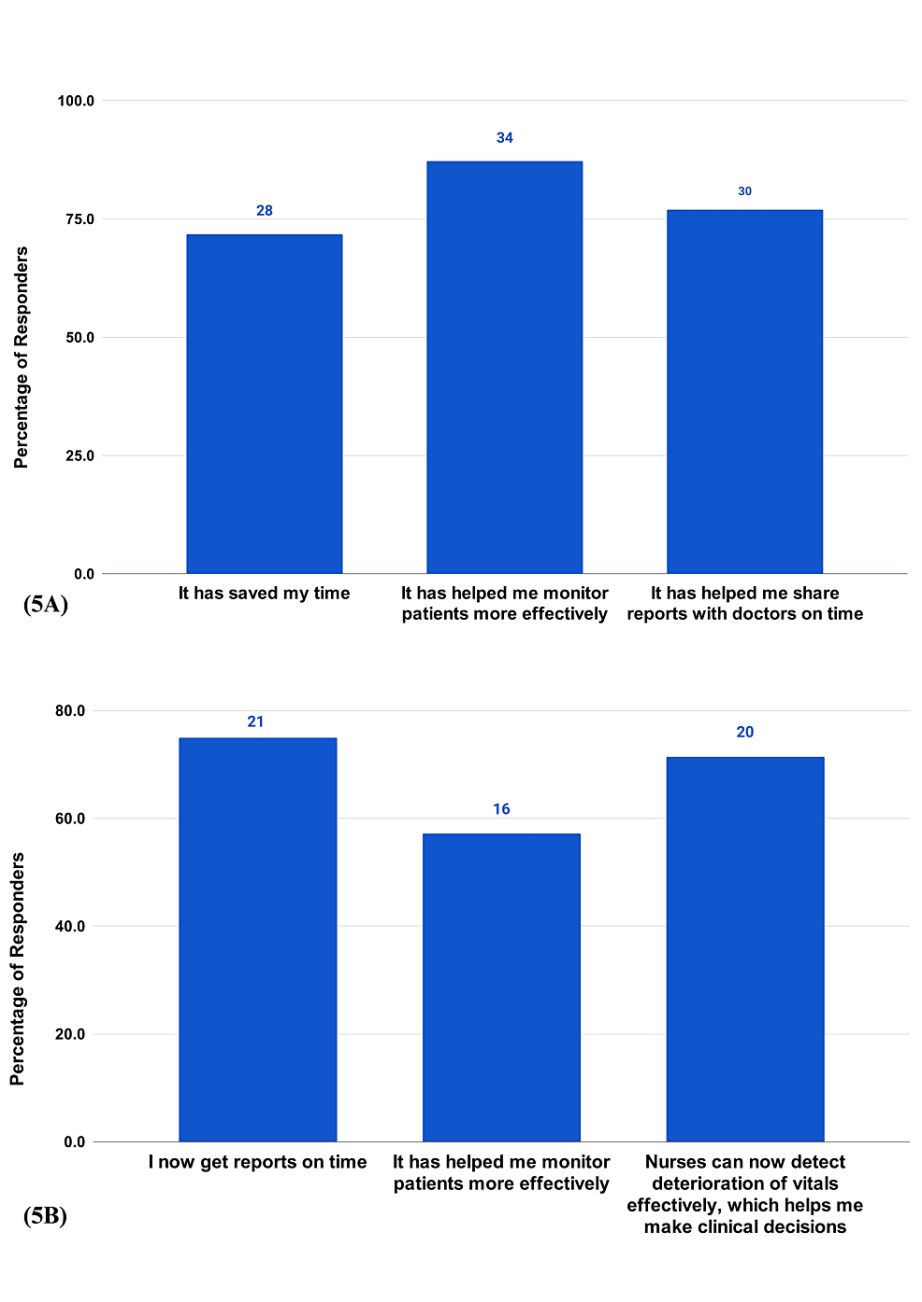
HCP survey questions on RPMS benefits 5A) How has automating and digitizing data using RPMS changed your workday? (Nurses (39)). 5B) How has automating and digitizing data using RPMS changed your workday? (Doctors (28)).

There is a high value placed on the early detection of patient deterioration, with 92.31% of nurses and 78.57% of doctors selecting this option (Figure 4). A balanced recognition of other benefits, like the improvement in patient outcomes, increase in patient safety, reduction in time spent on vitals monitoring, and the automation of data, was noted. A portion (74.4%) of nurses and 46.4% of doctors selected all the options (Figure 4). Benefits also extended to the HCP workday, with the majority of the respondents appreciating the comprehensive impact of the RPMS, in saving time, monitoring patients effectively, timeliness in communication among HCPs, effective patient monitoring, clinical decisions, and allowing nurses to detect deterioration effectively (Figure 5A, 5B).

Patient Safety and Care

**Figure 6:**
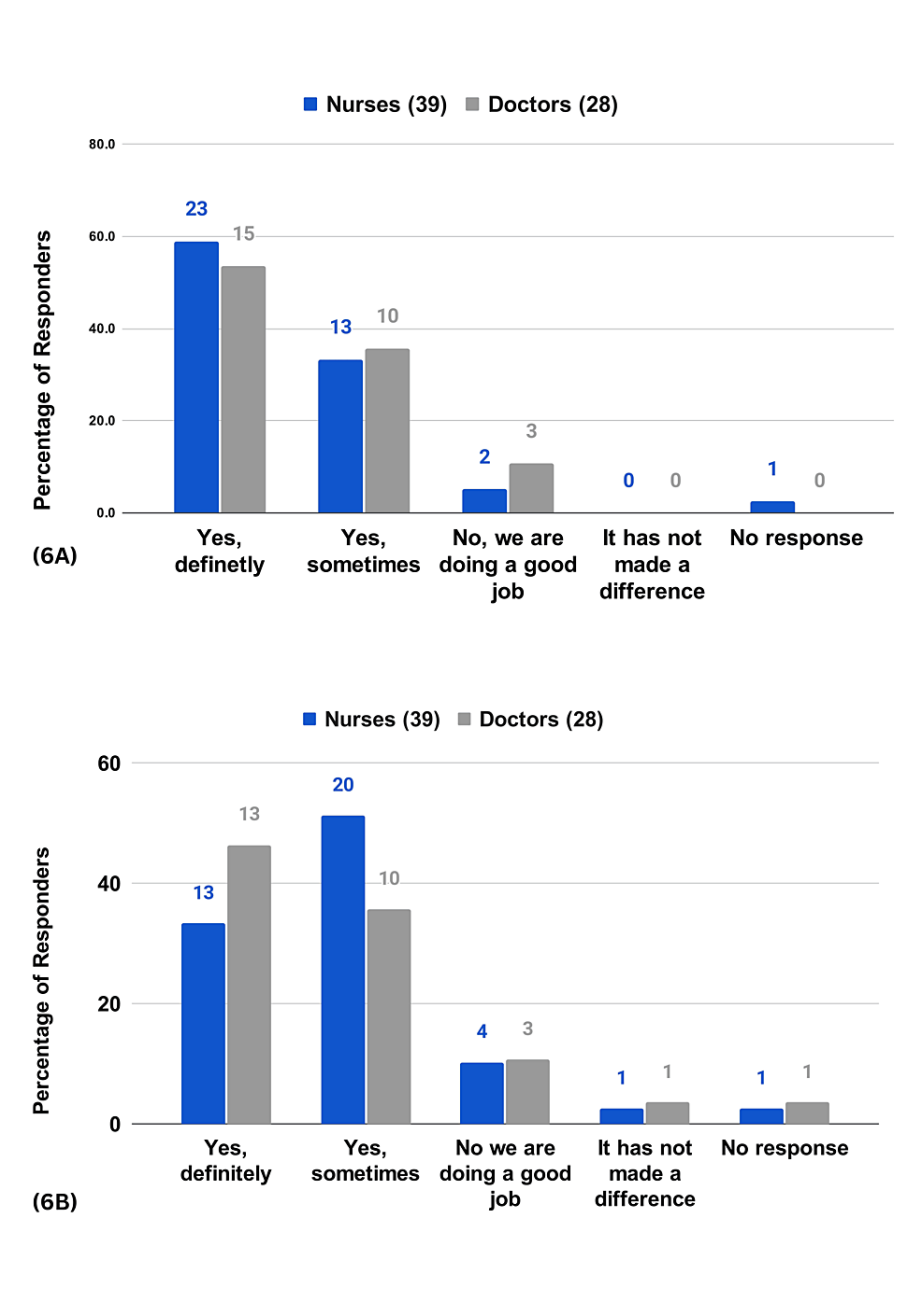
HCP survey questions on patient safety and care 6A) Has timely intervention of RPMS helped you achieve better patient outcomes? 6B) In your view, has the human error (due to workload) reduced, with RPMS’s use?

**Figure 7:**
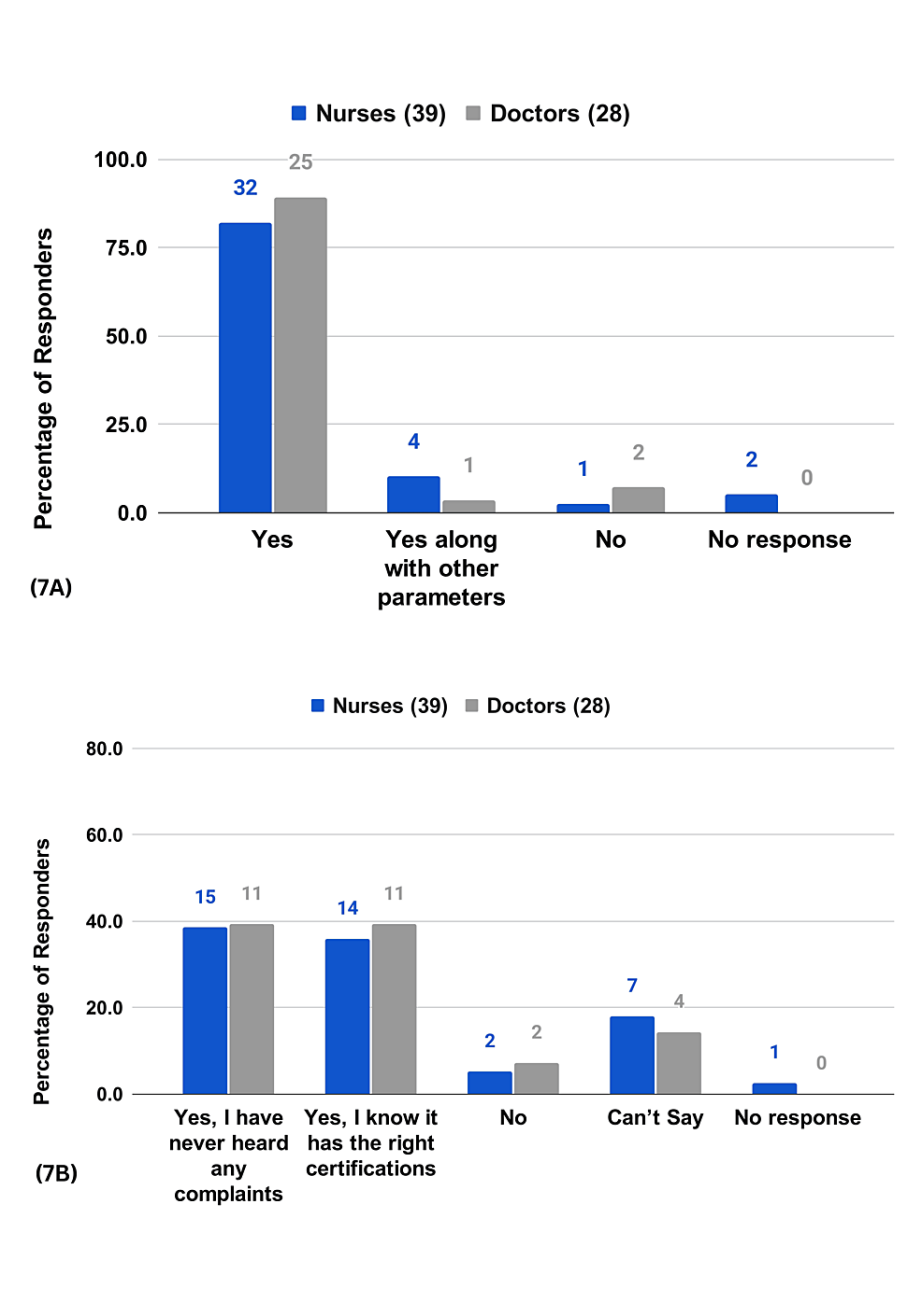
HCP survey questions on patient safety and care 7A) In your view, is RPMS's data trustworthy for you to make clinical decisions? 7B) In your experience do patients feel safe on RPMS?

**Figure 8:**
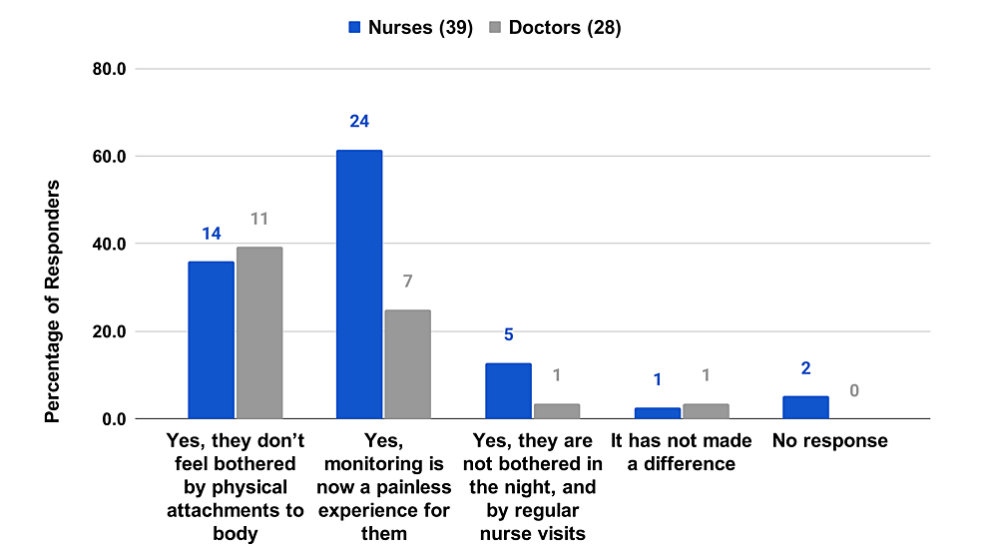
HCP survey questions on patient safety and care Patients' rest and well-being are crucial to recovery. Do you feel RPMS helps in that aspect?

A majority of the respondents (92.3% of the nurses and 89.3% of the doctors) agree that timely intervention through RPMS leads to better patient outcomes (Figure 6A). Many respondents (84.6% of the nurses and 82.1% of the doctors) agree that the system has reduced human errors due to workload (Figure 6B). A majority of the respondents (92.3% of the nurses and 92.8% of the doctors) believe the data from RPMS is trustworthy for clinical decisions (Figure 7A). Meanwhile, 74.3% of the nurses and 78.6% of the doctors largely believe that patients feel safe while on RPMS (Figure 7B). HCPs also acknowledge the positive impact of RPMS on patient rest and well-being, emphasizing the system's unobtrusiveness and the painless monitoring experience it provides (Figure 8).

Patient Experience

**Figure 9:**
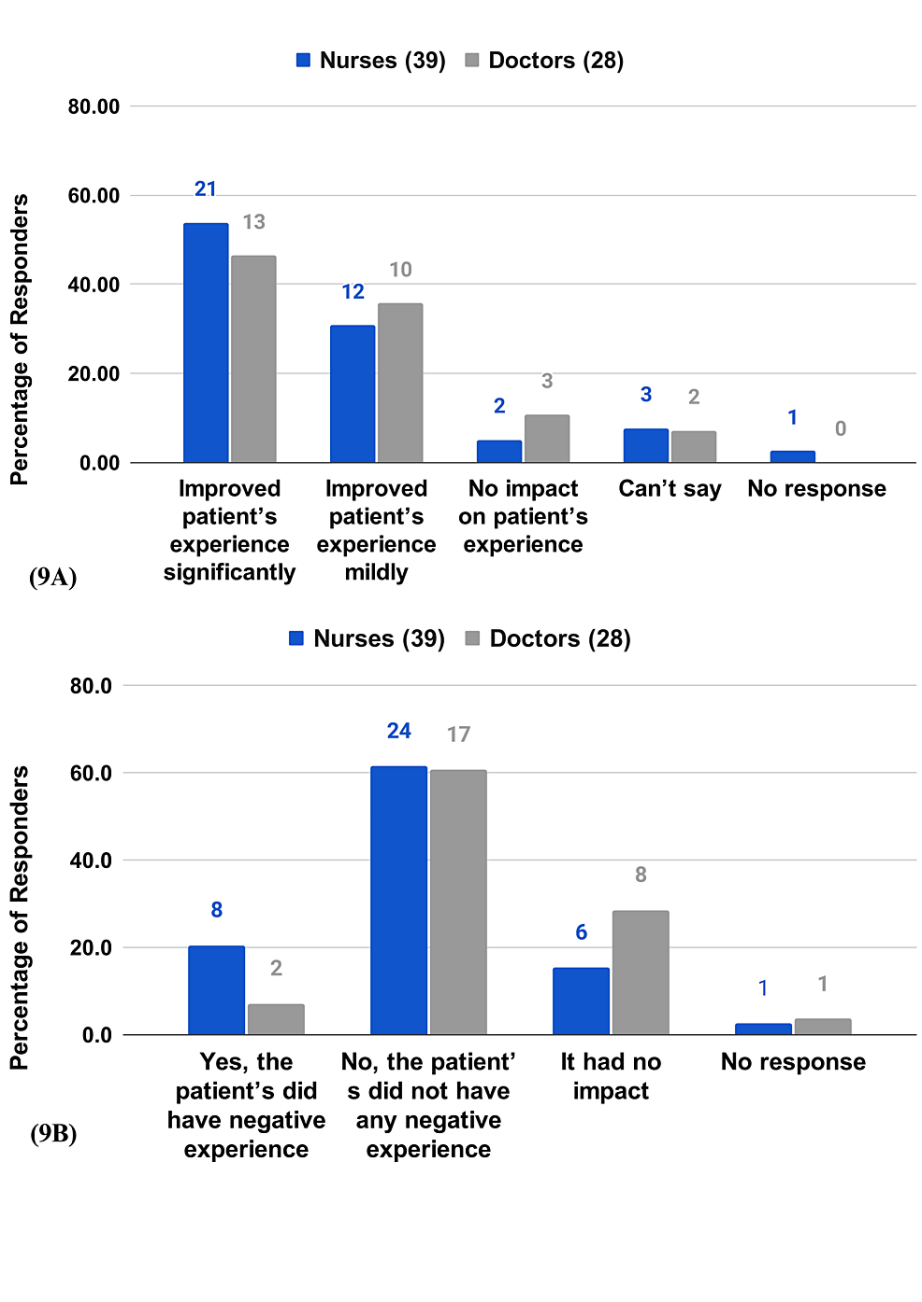
HCP survey questions on patient experience 9A) In your opinion, what impact does RPMS have on a patient's experience? 9B) Did RPMS have any negative impact on patient’s overall experience?

The majority of the respondents (84.6% of the nurses and 82.1% of the doctors) agree that the RPMS has improved patient experience(Figure 9A). More than half (61.5% of nurses and 60.7% of the doctors) stated that patients did not have any negative experience with RPMS (Figure 9B).

Ease of Use

**Figure 10:**
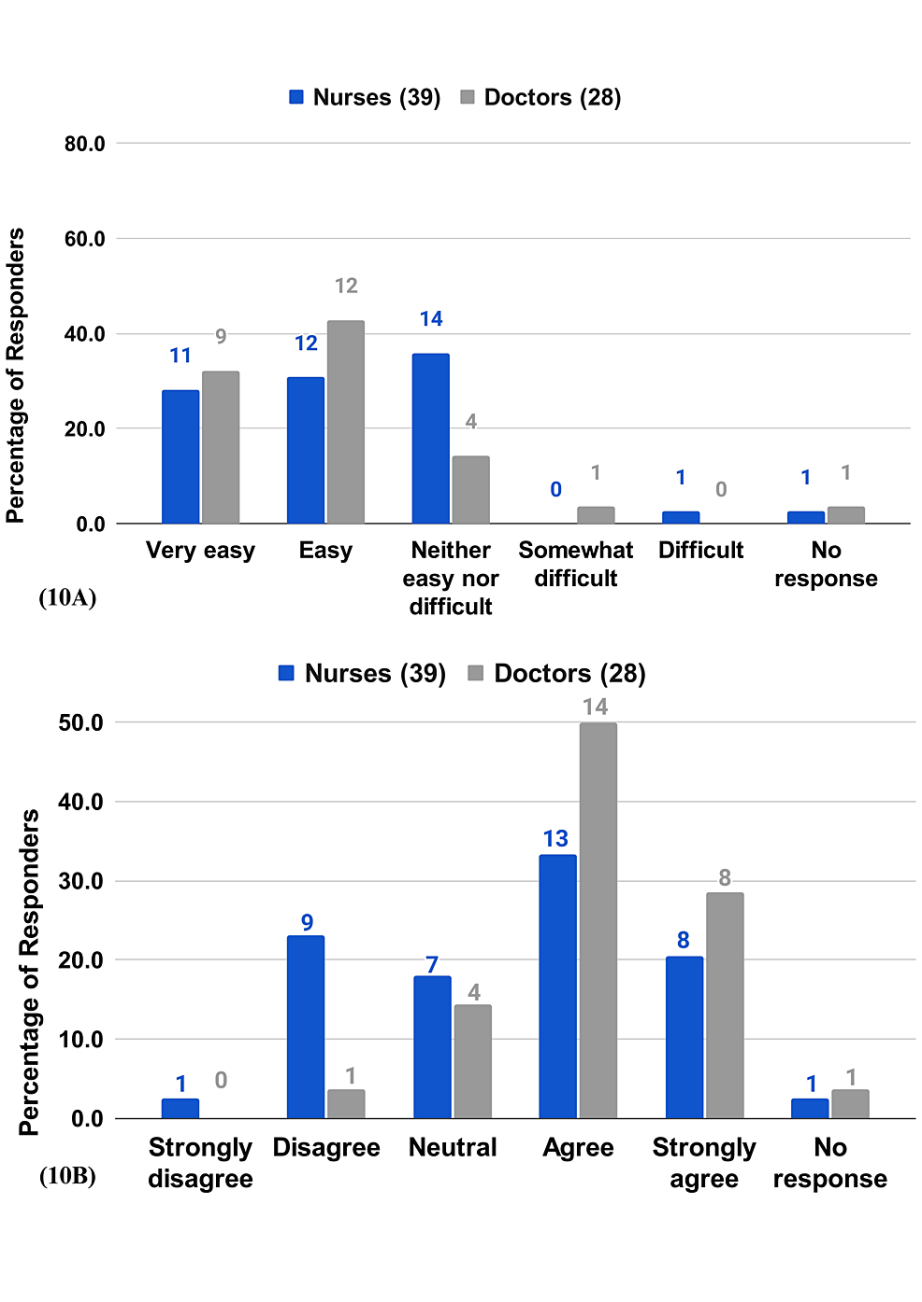
HCP survey questions on patients experience 10A) How easy or difficult is it to use RPMS system? 10B) RPMS is user-friendly.

**Figure 11:**
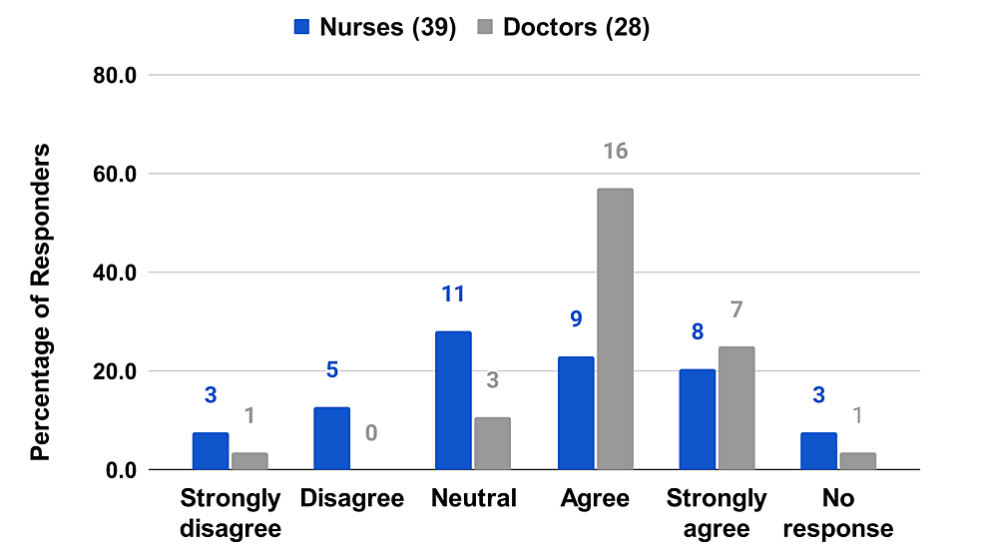
HCP survey questions on patient experience I learned to use it fairly quickly.

More than half of the nurses (60%) and 75% of the doctors find the RPMS either easy or very easy to use (Figure 10A). The majority of the respondents (53.8% of the nurses and 78.6% of the doctors) find the RPMS user-friendly (Figure 10B). A portion (43.5% of the nurses and 82.1% of the doctors) were able to learn to use the RPMS fairly quickly (Figure 11).

Satisfaction and Recommendation

**Figure 12:**
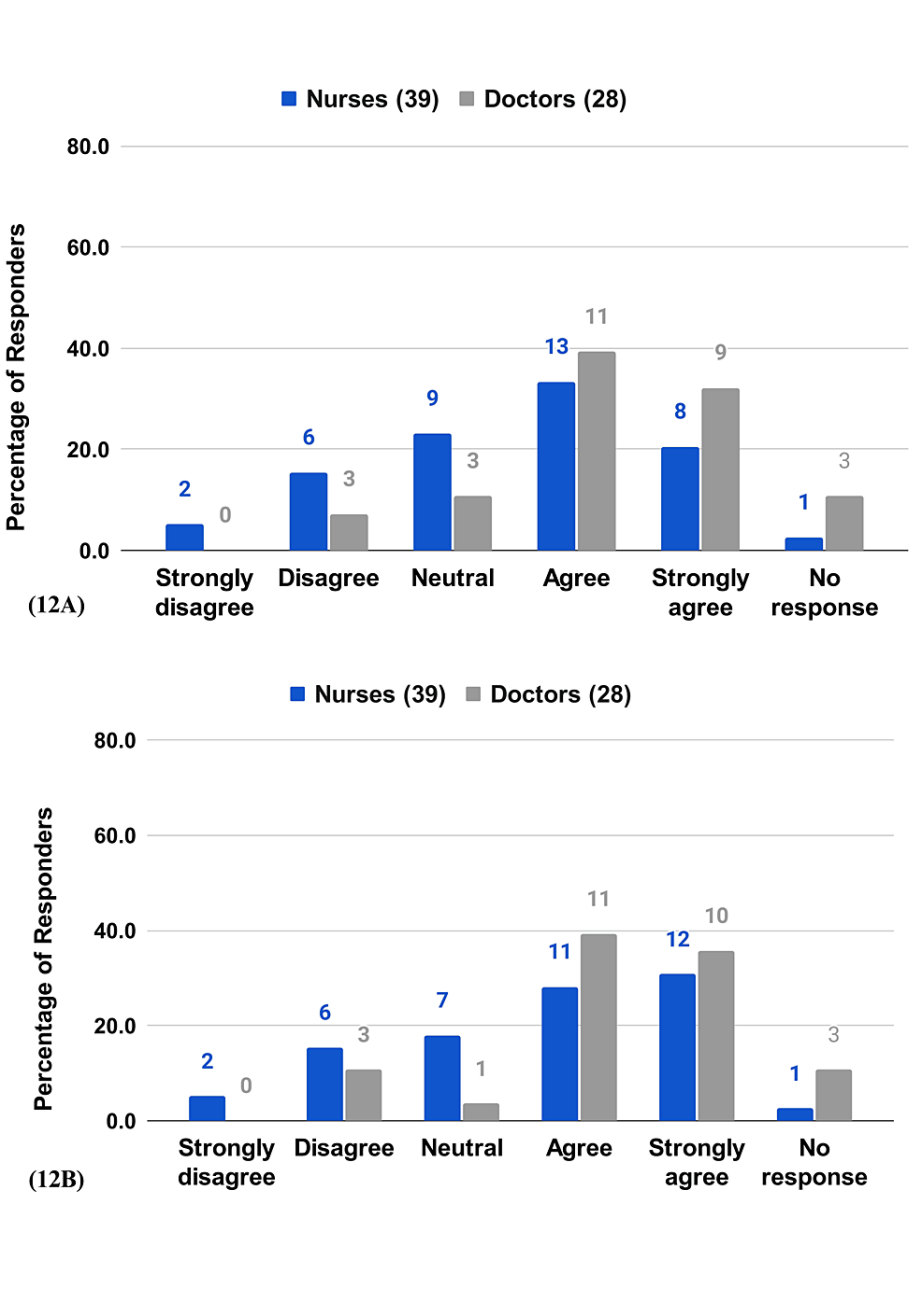
HCP survey questions on satisfaction and recommendation 12A) I am satisfied with RPMS. 12B) I would recommend the RPMS system to a colleague.

Satisfaction levels are high, with 53.8% of the nurses and 71.4% of the doctors agreeing or strongly agreeing. The willingness to recommend RPMS to peers is also high, with 60% of nurses and 75% of doctors agreeing or strongly agreeing (Figure 12A, 12B).

Patient feedback

**Figure 13:**
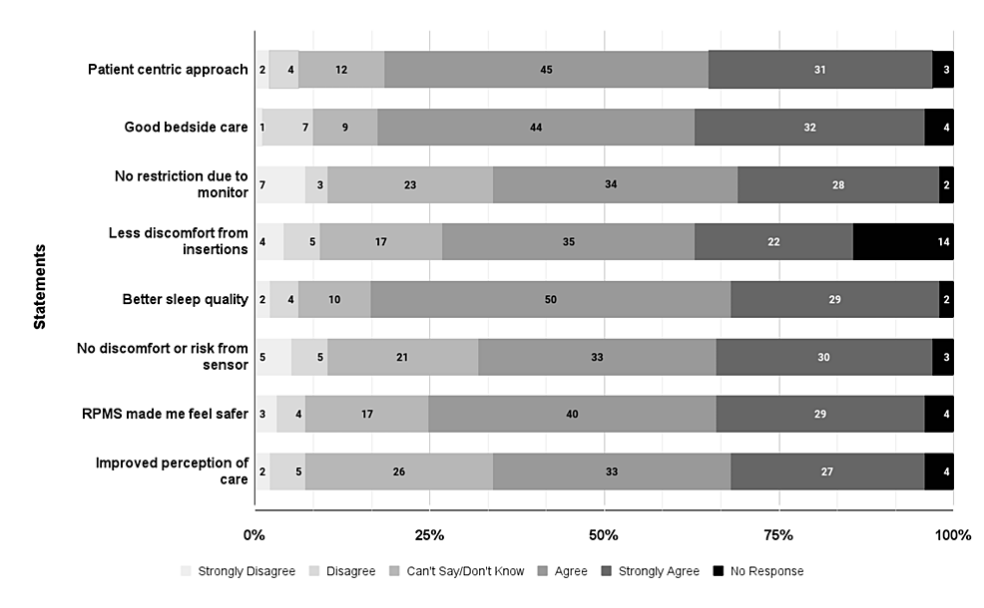
Patient survey questions

A survey conducted among 97 patients in wards equipped with RPMS revealed a positive reception toward the integration of this technology in hospital care, particularly influencing patients' perceptions of care, comfort, mobility, and safety. Many patients (81.9%) felt that the staff adopted a more patient-centric approach due to RPMS, and 81.7% reported enhanced bedside care, signifying a considerable improvement in nursing services. Regarding comfort and mobility, 65.3% of the patients did not feel restricted by the monitoring equipment, although 24.2% expressed neutral feelings. Only 26.5% strongly agreed that they experienced less discomfort from medical insertions compared to previous admissions, while 83.1% enjoyed better sleep quality, appreciating fewer nighttime disturbances. In terms of safety, 67% of the patients were comfortable with the sensor technology used, and 74.2% felt safer with the implementation of RPMS. Overall, 62.5% of the respondents observed an overall improvement in care quality since their last admission (Figure 13).

## Discussion

The deployment of RPMS in general wards signifies a positive shift in healthcare delivery in general wards. By facilitating continuous monitoring, RPMS has the potential to enhance both patient outcomes and clinical workflows. This study offers a comprehensive evaluation of RPMS, providing essential insights into its multifaceted impact on patient safety, nursing workflows, HCP satisfaction, and patient care quality, and how these elements contribute to the broader goals of modern healthcare systems.

This study has confirmed the patient-centric benefits of RPMS in general wards, particularly in enhancing patient safety and health outcomes-core components of patient-centered care. HCPs surveyed reported that RPMS has notably improved the patient safety and efficacy of monitoring. Central to the effectiveness of RPMS is its ability to provide continuous monitoring. Continuous monitoring has been shown to positively impact patient health, significantly reducing mortality rates and the duration of hospital stays [5,10-15]. Continuous monitoring facilitates the early detection of patient deterioration. This early detection is critical as it enables timely clinical interventions that are essential for preventing adverse events and improving clinical outcomes. HCPs surveyed have consistently recognized this feature as the most significant benefit of RPMS, underscoring its role in proactive patient care. These findings align with literature where HCPs emphasize the benefits of continuous vital sign monitoring in detecting clinical deterioration earlier [20-23]. 

Moreover, one of the foremost criteria for any patient monitoring technology is the reliability and accuracy of the data it provides. In this study, HCPs reported high levels of trust in the data generated by RPMS. This trust is crucial as it underpins the entire utility of the technology and directly impacts the clinical decisions made by doctors and nurses [24]. The reliable and accurate data from RPMS ensure that HCPs can make informed, timely decisions that are critical to patient care.

Importantly, both nurses and doctors acknowledged the system’s impact on reducing workload and human error [25]. Reduction in workload is not merely a procedural benefit, but it can also enhance job satisfaction and reduce burnout among healthcare staff. These findings are consistent with what is reported in the literature, underscoring the widespread benefits observed in similar studies [26].

In addition, the feedback from both HCPs and patients perceived an increase in the level of care provided to the patients. Most HCPs report that patients feel safer and more at ease while under RPMS monitoring. HCPs also note the positive impact of RPMS on patient rest and well-being, emphasizing the system's unobtrusive nature and the hassle free monitoring experience it offers. Such features are crucial for ensuring that the technology does not interfere with the patient's stay in the hospital or exacerbate their condition. These findings are in line with a growing body of research advocating for the adoption of patient-centered technologies [27]. Such technologies not only enhance care quality but also elevate patient safety across various healthcare settings. The consistent positive feedback from HCPs regarding RPMS underscores its profound impact on enhancing patient safety, affirming its effectiveness in real-world clinical settings, and reinforcing its vital role in the ongoing evolution of patient-centered healthcare technologies.

The time-motion study conducted provides compelling evidence that RPMS significantly enhances nursing efficiency in various crucial aspects. RPMS impacts nursing efficiency by enhancing patient care, simplifying communication, and reducing unnecessary workload. Primarily, the study demonstrated that nurses in RPMS-equipped wards were able to allocate more time to patient care activities. By automating routine tasks, such as vital checks, RPMS reduces the time nurses spend on these duties, allowing them to focus more on patient interactions. Similarly, a published study found that nurses reduced daily patient monitoring time by at least 49 minutes per patient, potentially freeing up additional time for activities that enhance patient wellbeing [28]. Notably, documentation during the night time in RPMS wards was higher than in control wards. This increase could be attributed to nurses having more time available for documentation due to the time saved by RPMS in other activities.

The ease of use of any new technology is crucial for its successful adoption in healthcare settings [24], where both the complexity of operations and the stakes of patient care are exceedingly high. Feedback from HCPs in this study underscores that RPMS was not only easy to use, easy to learn, but also integrated seamlessly with existing clinical practices. This seamless integration into daily routines reflects a well-considered system design that addresses practical aspects of healthcare delivery, such as the need for accessible, user-friendly interfaces, and minimal learning curves.

Finally, the acceptance and comfort of patients with RPMS were notably high. Patients reported a positive perception of continuous monitoring, feeling safer, and more cared for due to the perceived increase in attention. Importantly, the non-intrusiveness of RPMS technology contributed significantly to patient comfort, which is often a concern with traditional monitoring methods that may involve frequent disturbances and physical discomfort. The favorable patient feedback regarding reduced nighttime disturbances and the unobtrusive nature of the monitoring system underscore the importance of designing patient-centered technologies that prioritize patient comfort and trust [29]. This sense of security is vital for patient confidence in their treatment plan and overall satisfaction with the healthcare service.

While the findings predominantly highlight the benefits of RPMS, it is crucial to consider potential drawbacks and challenges associated with its implementations. These may include significant initial investment costs, the need for extensive staff training, potential resistance to change from HCPs, and technical reliability and accuracy issues. In addition, the risk of false alarms and alarm fatigue, challenges related to integrating the system with existing hospital infrastructure, concerns about data privacy and security, patient comfort and compliance, and the potential for data overload for HCPs are also important considerations [30]. Addressing these challenges is essential to ensure the successful adoption and sustainability of RPMS in healthcare settings.

This study has some limitations. Conducted in a single hospital with a specific group of nurses and doctors, the findings may not be generalizable to other healthcare settings with different operational dynamics and patient demographics. The study methods might have to be altered to fit the specific health care settings. In addition, the feedback from HCPs and patients is subjective and could be influenced by personal biases or expectations. Moreover, the use of self-reported data may introduce bias, as participants could overstate benefits or underreport issues due to perceived expectations from the research team. Confounding variables such as HCP experience levels, patient acuity, and ward staffing ratios likely influenced the results.

## Conclusions

This study has demonstrated that the deployed RPMS in MSR significantly improved the quality of care provided in general wards by enhancing patient safety, reducing the workload on HCPs, and streamlining communication and documentation processes. Key findings include substantial reductions in the time spent on routine tasks, such as vitals checks and documentation, allowing nurses more time for direct patient care and interaction. Furthermore, both HCPs and patients reported high levels of satisfaction with the system, noting improved safety, communication, and overall care quality. Further studies are needed to quantitate the patient benefits, the cost-effectiveness of the system and should explore similar implementations in other hospitals to build on these findings.

## Figures and Tables

**Figure 14:**
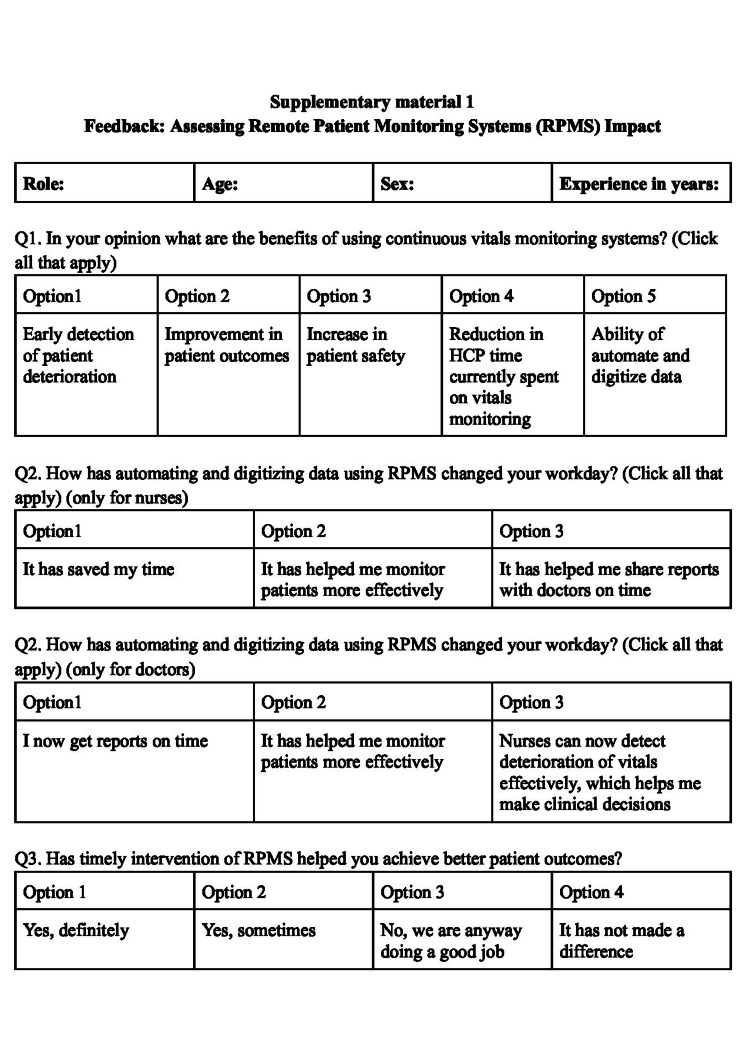
HCP survey questions 1-3.

**Figure 15:**
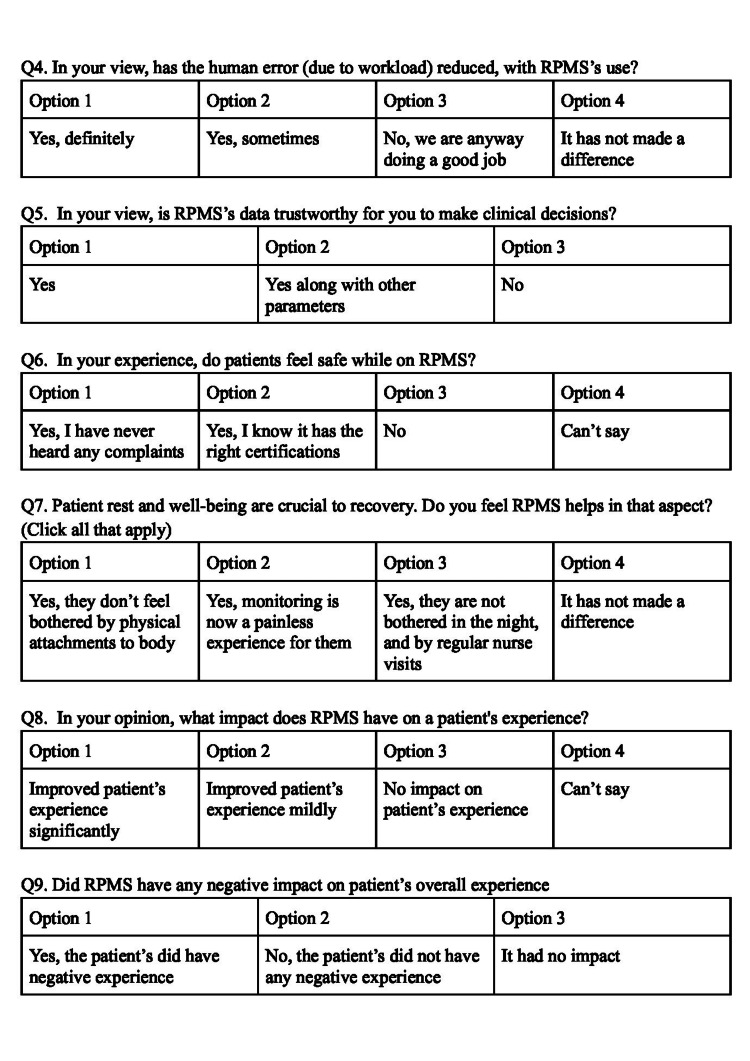
HCP survey questions 4-9.

**Figure 16:**
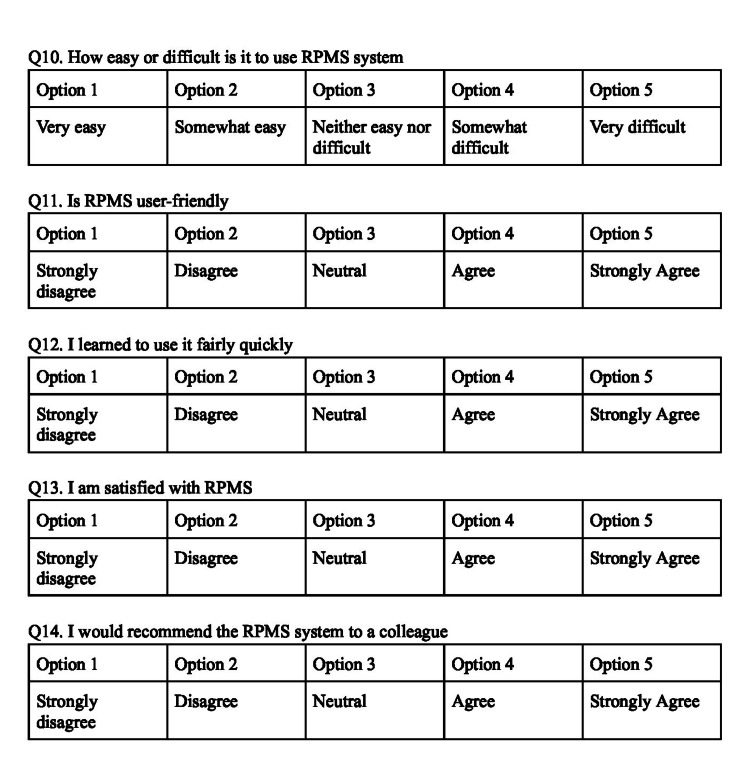
HCP survey questions 10-14.

**Figure 17:**
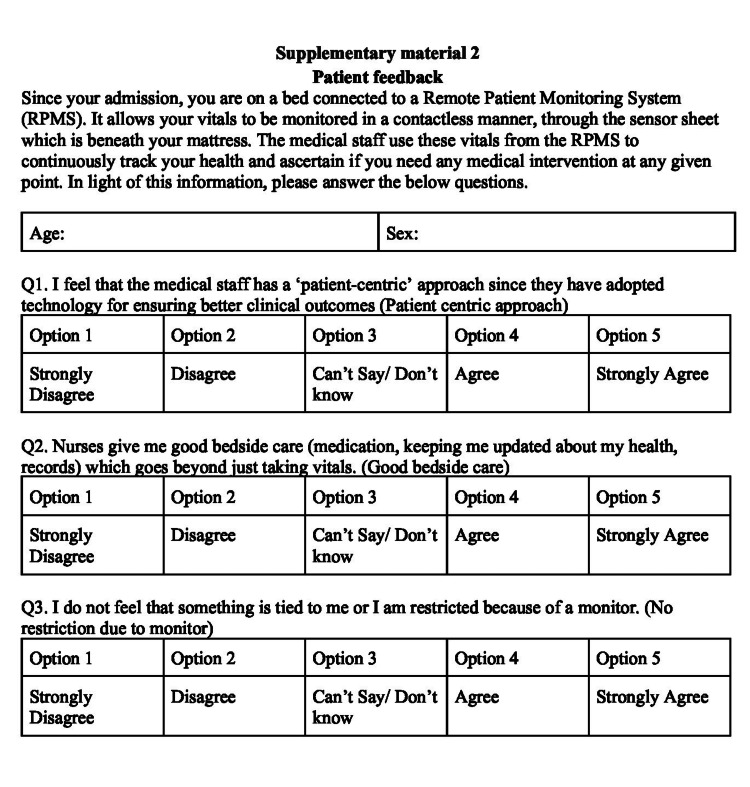
Patient survey questions 1-3.

**Figure 18:**
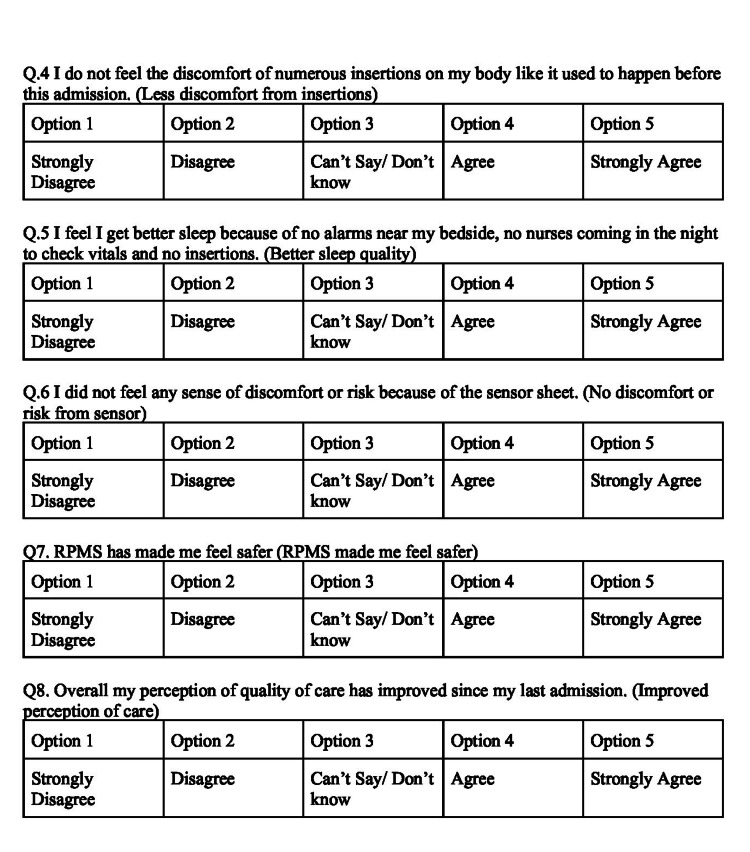
Patient survey questions 4-8.

## Appendices

Appendix A

Appendix B
